# Anandamide-Induced Neuroprotection of Cortical Neurons Relies on Metabolic/Redox Regulation and Mitochondrial Dynamics

**DOI:** 10.1007/s12035-025-05514-z

**Published:** 2025-11-24

**Authors:** Ana Laura Torres-Román, Tània Gavaldà-Vives, Samuel Simón-Sánchez, Omar Emiliano Aparicio-Trejo, José Pedraza-Chaverri, Tessy López-Goerne, Alette Ortega Gómez, Alexey A. Tinkov, Michael Aschner, Ismael Galve-Roperh, Abel Santamaría

**Affiliations:** 1 Programa de Posgrado (Doctorado) en Ciencias Biológicas, Universidad Nacional Autónoma de México, 04510 Mexico City, Mexico; 2 Departamento de Bioquímica y Biología Molecular, Instituto Universitario de Investigación en Neuroquímica (IUIN), Universidad Complutense, 28040 Madrid, Spain; 3 Instituto de Investigaciones Sanitarias Ramón y Cajal y Centro de Investigaciones en Red Enfermedades Neurodegenerativas, Madrid, Spain; 4 Department of Cardio-Renal Physiopathology, Instituto de Cardiología Ignacio Chávez, 14080 Mexico City, Mexico; 5 Laboratorio F-315, Departamento de Biología, Facultad de Química, Universidad Nacional Autónoma de México, 04510 Mexico City, Mexico; 6 Laboratorio de Nanotecnología y Nanomedicina, Departamento de Atención a La Salud, Universidad Autónoma Metropolitana-Xochimilco, 04960 Mexico City, Mexico; 7 Subdirección de Oncología y Hematología, Instituto Nacional de Cancerología, 14080 Mexico City, Mexico; 8 Department of Medical Elementology, Peoples Friendship University of Russia, RUDN University), Moscow, 117198 Russia; 9 Center of Bioelementology and Human Ecology, IM Sechenov First Moscow State Medical University (Sechenov University), Moscow, 119146 Russia; 10 Laboratory of Molecular Ecobiomonitoring and Quality Control, Yaroslavl State University, Yaroslavl, 150003 Russia; 11 Department of Molecular Pharmacology, Albert Einstein College of Medicine, Bronx, NY 10461 USA; 12 Facultad de Ciencias, Universidad Nacional Autónoma de México, 04510 Mexico City, Mexico

**Keywords:** Endocannabinoid System, Anandamide, PPARγ receptor, Cannabinoid receptors, Energy metabolism, Mitochondrial dynamics

## Abstract

Mitochondrial disruption is a key mechanism in the etiology of neurodegenerative diseases. Promoting mitochondrial dynamics and renewal of the mitochondrial network can restore its function and sustain neuronal viability. Although a growing body of evidence implicates endocannabinoid signaling in the regulation of mitochondrial function, its neuroprotective role in neurodegenerative diseases remains largely unexplored. Clarifying this relationship is crucial for understanding the therapeutic efficacy of the endocannabinoid system. This study aimed to evaluate whether endocannabinoid signaling via PPARγ and CB_1_ receptors regulates mitochondrial biogenesis and dynamics, exerting neuroprotective actions. Primary cortical neuronal cultures were subject to energy deficiency and excitotoxicity with 3-nitropropionic acid (3NP) and quinolinic acid (QUIN). Neurons were pretreated with the endogenous cannabinoid anandamide (AEA 100 nM), and cell viability and lipid peroxidation levels were characterized. To further explore mitochondrial status, immunofluorescence, western blot, and qPCR of mitochondrial proteins or genes were carried out. The metabolic status was assessed by oxygen consumption and extracellular acidification rates. Intracellular calcium levels and PPARγ transactivation were also analyzed. 3NP + QUIN induced neuronal damage, while AEA treatment afforded a neuroprotective effect. The use of selective receptor antagonists indicated that AEA neuroprotection depends on both PPARγ and CB_1_ receptors. AEA also increased mitochondrial biogenesis, fission markers and OXPHOS function, while delayed Ca^2+^ levels and induced PPARγ transactivation. In conclusion, AEA afforded neuroprotection secondary to increased mitochondrial biogenesis and redox regulation triggered by the activation of CB_1_ and the nuclear receptor PPARγ.

**Supplementary Information:**

The online version contains supplementary material available at 10.1007/s12035-025-05514-z.

## Introduction

Neurodegenerative diseases (NDs) are characterized by progressive degeneration of neurons, resulting in irreparable brain damage, and include Alzheimer’s disease (AD), Parkinson’s disease (PD), Huntington’s disease (HD) and amyotrophic lateral sclerosis (ALS). All are characterized by mitochondrial dysfunction as a central pathogenic event that contributes to both the onset and progression of the disease [1]. In NDs, energy deficiency and excitotoxicity interfere with the optimal functioning of neuronal circuits, often culminating in neuronal cell death. Excitotoxicity involves a series of events triggered by persistent glutamate activity and overstimulation of N-methyl-D-aspartate (NMDA) and α-amino-3-hydroxy-5-methyl-4-isoxazolepropionic acid (AMPA) receptors, leading to an imbalance in Ca^2+^ homeostasis. Under physiological conditions, mitochondria in the perisynaptic region, particularly in excitatory neurons, internalize calcium to regulate homeostasis; however, under conditions of Ca^2+^ overload, the mitochondrial membrane potential collapses, leading to energy deficiency, oxidative stress and activation of apoptotic and necrotic cell death pathways [2]. Accordingly, targeting mechanisms of mitochondrial preservation in excitotoxic events could favor the preservation of neuronal integrity in NDs [3].

The central nervous system (CNS) is particularly vulnerable to mitochondrial damage due to its high metabolic activity and the postmitotic nature of neurons, which favors the accumulation of reactive oxygen species (ROS), as often observed in aged brains. Mitochondrial damage and respiratory chain (OXPHOS) dysfunction can originate from ROS-induced mutations of mitochondrial genes (either encoded in mitochondrial and nuclear DNA) or by the aggregation of misfolded proteins that interfere with the functioning of mitochondrial complexes [4]. Neurons are highly polarized cells with a high energy consumption which is met primarily by oxidative phosphorylation of mitochondria, which constantly undergo fusion and fission, altering their morphology and function, metabolic demand, and stress [5]. These dynamic processes are regulated among others by the GTPases, dynamin-related protein 1 (DRP1), which directs fission, and optic atrophy type 1 (OPA1) and mitofusins 1/2 (Mfn1/2), which regulate fusion [6, 7]. In this regard, Peroxisome Proliferator-Activated Receptor Gamma (PPARγ), a nuclear receptor and transcription factor, plays a significant role in regulating neuronal mitochondrial dynamics, as the activation of this receptor increases mitochondrial DNA (mtDNA) expression and ATP levels in in vitro and murine models of NDs [8]. PPARγ activation, and its association with the coactivator PGC1-α, leads to increased mitochondrial biogenesis through coordinated transcription between nuclear and mitochondrial DNA mediated by mitochondrial transcription factor A (TFAM) [9, 10]. In turn, mitochondrial biogenesis improves mitochondrial function and contributes to neuronal survival and protection against NDs. Preclinical studies support the potential use of PPAR-targeted agonists and mitochondrial restoration as an effective treatment for NDs [11, 12].

The Endocannabinoid System (ECS) is a neuromodulatory system that has been widely studied for its neuroprotective potential, mainly through antioxidant and anti-inflammatory mechanisms triggered after the activation of cannabinoid receptor 1 (CB_1_) [13, 14]. Emerging evidence highlights the mechanistic links between the endocannabinoid system (ECS) and cellular bioenergetics, particularly via the activation of cannabinoid receptor type 1 (CB_1_), mitochondrial CB_1_ receptors (mtCB_1_), and peroxisome proliferator-activated receptors (PPARs) [15]. Endocannabinoids (ECs) are known to modulate the redox balance both under physiological and stress conditions [16]. In addition, cannabinoids upregulate proteins involved in mitochondrial biogenesis and dynamics in a process mediated by PPARγ and CB_1_ receptors [17–19]. Moreover, the presence of CB_1_ receptors in mitochondria has emerged as an alternative mechanism for direct regulation of mitochondrial function [20–22]. In this regard, several cannabinoid agonists have been shown to regulate mitochondrial dynamics, mainly phytocannabinoids, though the potential role of ECs cannot be ruled out [23].

Anandamide (N-arachidonoylethanolamine or AEA) is an endogenous cannabinoid agonist that exhibits neuroprotective effects via CB_1_ activation, regulates the redox neural balance, and exerts mitochondrial modulatory properties, as well as efficacy as a PPARγ receptor agonist [24, 25]. In this regard, the role of mitochondrial dynamics regulation by cannabinoid signaling as a mechanism responsible for its neuroprotective effect has yet to be fully characterized. Therefore, this study aims to evaluate whether stimulation of the ECS by AEA can regulate mitochondrial biogenesis and dynamics through the PPARγ/PGC1α pathway to exert neuroprotective actions in a model of neurodegeneration combining energy deficiency and excitotoxicity in neuronal cells.

## Materials and Methods

### Cell Culture

Primary neuronal cultures were obtained from the cortex of E14 C57BL/6 mice using the papain dissociation system kit (Worthington-Biochem). The cells were grown in Neurobasal growth medium supplemented with 2% B-27 and plated in culture dishes pretreated overnight with poly-L-lysine. Cells were cultured in 48, 24 or 6 well culture plates and incubated at 37 °C in a 5% CO₂ atmosphere. Experimental procedures were performed in accordance with the guidelines and approval of the Animal Welfare Committees of Universidad Complutense de Madrid, Comunidad de Madrid, the directives of the Spanish Government and the European Commission (RD 118/2021). Animals had unrestricted access to food and water. They were housed (typically, 4–5 mice per cage) under controlled temperature (range, 20–22 °C), humidity (range, 50–55%), inversed light/dark cycle (12 h/12 h) and with environmental enrichment.

### Treatments

A model of neurodegeneration combining two of the most relevant characteristics of NDs: excitotoxicity and energy deficiency triggered by mitochondrial damage, was developed. Two neurotoxins were administered to the cultures simultaneously: 3-nitropropionic acid (3NP), described as a selective inhibitor of the mitochondrial enzyme succinate dehydrogenase (SDH, Complex II of the electron transport chain) which promotes oxidative damage and subsequent neuronal death [25], and quinolinic acid (QUIN), an excitotoxin acting in the CNS and agonist of the glutamatergic NMDA-type receptor [26, 27]. Primary neuronal cultures were pretreated 6 h before with 100 nM AEA, this being the lowest effective concentration observed in a dose–response viability assay and then exposed to 3NP (0.5 mM) + QUIN (50 μM) [28–30] for an additional 24 h. The PPARγ receptor antagonist GW9662, and the CB_1_ antagonist SR141716, were added to the cultures 1 h before receiving the cannabinoid treatments, both at the concentration of 0.5 µM.

### Cell Viability Assay

Cell viability was calculated by the conversion of 3-(4,5-dimethylthiazol-2-yl)−2,5-diphenyltetrazolium bromide (MTT) to formazan as an indirect indicator of mitochondrial activity. MTT was prepared in deionized water (1 mg/ml) and 50 µl was added to each well in a 24-well plate. Subsequently, all plates were incubated for 3 h at 37 °C in an atmosphere of 5% CO_2_. Next, MTT reduction was quantified spectrophotometrically at 595 nm in the iMark microplate ELISA reader (Bio-Rad, Ca, USA). Absorbance data were normalized against cell number for each treatment.

### Determination of ATP Levels

The CellTiter-Glo® luminescent cell viability kit was used to quantify the levels of ATP present. For this purpose, 250 μl of CellTiter-Glo® reagent was added directly to cells cultured in 24-well plates with 250 μl of neurobasal medium, and moderately resuspended to promote cell lysis and the release of the ATP present. The plate was incubated for 15 min, considering the same reagent incubation times for each well, and the luminescence was subsequently quantified in a Luminescence Spectrometer Fluostart Omega BMG Labtech.

### Lipid Peroxidation Assay

Lipid peroxidation was assessed with the thiobarbituric acid-reactive substances (TBARS) assay, which is based on the colorimetric change caused by the reaction of thiobarbituric acid (TBA) with malondialdehyde, one of the most abundant products of lipid peroxidation. Following cell treatments, homogenates were obtained, 100 μL of TBA reagent was added, and cells were incubated in a water bath at 94 °C for 20 min. Cells were then placed on ice for 5 min and centrifuged. The pellet was discarded, and the supernatants were analyzed for absorbance at 532 nm. A tetramethoxypropane (TMPO) standard curve was prepared, and a linear regression was constructed on this curve to obtain the equation of the straight line, yielding the result in ng of TMPO. These values ​​were correlated with the mg of protein levels ​​quantified for each sample using the Bradford method.

### Protein Expression

The key component of mitochondrial and neuronal function, the voltage-dependent anion channel (VDAC), was assessed by western blot analyses. Cell cultures seeded in 6-well plates were homogenized in lysis buffer (25 mM Tris, 50 mM NaCl, 2% Igepal, 0.2% SDS, and protease inhibitors) at pH 7.4. Protein concentration was determined by Bradford analysis [31]. Sixty micrograms of total protein were separated on a polyacrylamide gel (10%) and transferred to PVDF membranes incubated overnight in the presence of primary antibodies. Afterwards, membranes were washed 3 times and incubated for 45 min with the anti-mouse secondary antibody. GAPDH and β-actin were quantified and used as loading controls, upon availability. Immunoblots were performed in triplicate.

### Gene Expression

To evaluate whether AEA stimulation directs the expression of genes related to mitochondrial biogenesis in cultures, real time quantitative qPCR was carried out with primers specific to each gene. Total RNA was extracted with Nucleozol (Macherey–Nagel) and following the extraction protocol suggested by the manufacturer. RNA samples (1–2 µg) were subjected to reverse transcription (RT) using the Transcriptor First Strand cDNA Synthesis Kit (Roche Life Science) with random hexamer primers. Real time (RT)-qPCR was performed in 96 or 384-well plates using the LightCycler® Multiplex DNA Master (Roche Life Science) using Fast SYBR Green probe (Applied Biosystems #4,385,610) and appropriate primers in a 7900 HT-Fast (Applied Biosystems). 384 multiwell plates were analyzed in QuantStudio 12 K Flex and QuantStudio 7 Flex, respectively (Applied Biosystems). The 2^(-ΔΔCt) method was utilized to compute the relative expression ratio of the target gene in comparison to the reference gene. Determinations were performed at least in triplicates. A set of GAPDH/actin-specific primers was added to the PCR as control housekeeping genes. Sequences of the primers used are shown in Supplementary Table S.1.

### Immunofluorescence

To determine the increase in the number of mitochondria, the expression of the mitochondrial protein ATPβ was evaluated by immunofluorescence. Neurons were cultured on coverslips for 24 h, fixed with 4% paraformaldehyde/PBS for 15 min, and washed three times with PBS. Samples were blocked with 5% BSA for 60 min and permeabilized with PBS-Triton 0.25%. Culture samples were then incubated with or without primary mouse monoclonal antibody against ATPβ (dilution 1:500) and primary rabbit monoclonal antibody against β-tubulin as a neuronal marker (dilution 1:500) in PBS-Triton 0.25% overnight at 4 °C. Samples were then rinsed three times with PBS and incubated with their respective secondary antibody (dilution 1:2000) for 60 min at room temperature, and finally were incubated with DAPI for 15 min. After rinsing, the preparations were mounted and observed under the TCS SP8 laser scanning confocal microscope with Hyvolution super-resolution system, obtaining images of five randomly selected areas of each sample.

### MitoTracker Labeling

To label mitochondria, the fluorometric dye MitoTracker RedCMXRos (Thermo Fisher Cat. M7513) [32] was used at a concentration of 200 nM, prepared in complete growth medium and incubated for 15 min at 37 °C after the corresponding treatments. Subsequently, the culture medium was removed, and a careful wash was performed with tempered PBS. Then, cells were fixed with 3.7% formaldehyde in complete growth medium at 37 °C for 15 min and permeabilized in PBS containing 0.2% Triton X-100. The fluorescence was analyzed with a scanning confocal microscope at a wavelength of 579 nM (Carl Zeiss).

### Mitochondrial Membrane Potential (ΔΨM) Evaluation

After being treated, neurons were incubated with digitonin (5 μg/ul, 30 min) to permeabilize the cell membrane without altering the properties of the mitochondrial membrane. Subsequently, cells were incubated for 15 min with the fluorescent probe DiSC3 (5) [250 nM] (Cat. 11,520,286, Invitrogen). DiSC3(5), a lipophilic dye that accumulates in hyperpolarized membranes reflecting mitochondrial depolarization [33]. The fluorescence emission was monitored at an excitation wavelength of 635 nm and an emission spectrum of 620 to 700 nm, taking one measurement per second until completing 80 s with the Luminiscence Spectometer SLM-AMINCO.

### Assessment of Cellular Metabolism

Oxygen consumption rate (OCR) and extracellular acidification rate (ECAR) were determined using a Seahorse XF HS Mini Analyzer (Agilent Technologies, Inc., Waldbronn, Germany). Neurons were seeded in a Seahorse XF HS Mini Analyzer 6-well plate at a density of 15,000 cells per well, and following 7 DIV, cells were incubated for 30 min or 6 h with 100 nM AEA, and subsequently, with 3NP + QUIN for an additional 30 min or 24 h, respectively. To initiate the Seahorse XF assay, the following compounds from the Seahorse XF Cell Mito Stress Test kit (#133,015–100, Agilent Technologies, Inc.) were added to the wells at final concentrations, according to the instructions of the manufacturer; port A: 1 µM oligomycin (ATP synthase inhibitor), port B: FCCP 0.6 and 0.4 μM (carbonyl cyanide-4 (trifluoromethoxy) phenylhydrazone, an uncoupling agent that collapses the proton gradient and alters mitochondrial membrane potential), port C: 0.5 μM rotenone (Rot) (complex I inhibitor) and 0.5 μM antimycin A (AA) (inhibitor of complex III). Both OCR and ECAR were normalized per well, based on the number of cells determined after the experiment. Cells were fixed and photomicrographs were taken with an inverted microscope. Images were analyzed using Fiji/Image J software to assess the cell counting per treatment.

Using the OCR and ECAR data, basal respiration graphs were obtained by subtracting the OCR value when complexes I and III were inhibited (when treated with rotenone and/or antimycin A) from the initial OCR value (without any treatment with the complex inhibitors). ATP production was calculated by subtracting the OCR value when complex V was inhibited (when treated with oligomycin) from the initial OCR value. Maximum respiration corresponds to the value obtained when treated with FCCP. Reserve respiratory capacity corresponds to the OCR value when treated with FCCP minus the initial OCR value.

To obtain the graphs on the metabolic functionality of different experimental groups, the following calculations were carried out:

- Basal respiration = initial OCR values ​​- OCR values ​​after Rot and AA administration
- ATP production = initial OCR values ​​- OCR values ​​after oligomycin administration
- Maximal respiration = initial OCR values ​- OCR values ​​after FCCP administration
- Spare respiratory capacity = values ​​after FCCP administration—initial OCR values
- Basal glycolysis = initial ECAR values before any metabolic inhibition
- Compensatory glycolysis = ECAR values after Rot and AA administration

### Calcium Signaling Assay

To measure real time cytosolic calcium concentrations, we used the non-fluorescent Fluo-4 acetoxymethyl ester (Fluo-4 AM; Thermo Fisher Scientific) [34]. Neurons seeded in a 48-well plate were washed with PBS and subsequently incubated for 5 min with 2 µM Fluo-4AM in Hanks’ Balanced Salt Solution (HBSS) without calcium at 37ᵒC in a 5% CO_2_ atmosphere covered from light. After this time, 5 gentle washes were performed with HBSS medium without calcium. In the last wash, HBSS medium with calcium was added and incubated for an additional 15 min at 37 degrees in a 5% CO_2_ atmosphere covered from light. Fluorescence was measured with a fluorescence microscope with excitation and emission wavelengths of 485 nm and 528 nm, respectively. Treatments were added at a volume of 50 μL to each well, and measurements continued for 20 s per well. Images and videos were obtained during the addition of the treatment, except in the case of the AEA pretreatment for 15 min and 3 h, where images were obtained only during the addition of 3NP + QUIN. 3NP [2.5 mM] and QUIN [250 µM] were used to quantify calcium mobilization. Fluorescence intensity of Fluo4-AM was quantified for each image and the background value subtracted.

### PPARγ Transcriptional Assays

To study PPARγ transcriptional activity in the presence of AEA and neurotoxins, prior to seeding, cortical neurons were co-nucleofected with the GAL4-PPARγ construct and the luciferase reporter vector GAL4-luc using the Mouse Neuron Nucleofector Kit (Lonza, Cat No:VPG-1001) following the manufacturer's instructions, at a concentration of 3 μg of plasmid per each 3x10^6^cells, and seeded in 24-well plates at a density of 200,000 cells/cm^2^ in neurobasal medium supplemented with 2% B-27. After 48 h, cells were treated with vehicle (DMSO), 1 µM pioglitazone (PIO) as a positive control for PPARγ activation, 100 nM AEA and 200 nM AEA for 5 h, and 3NP + QUIN for an additional 6 h. At the end of treatments, cell extracts were collected in 20 µl of passive lysis buffer per well. To determine luciferase activity as a reporter protein for PPARγ transcriptional activity, the extracts were analyzed in a 96-well white plate, using the Dual-Luciferase Reporter System (Promega, Madison, WI, USA) kit [35]. Plates were quantified in the FLUOstar® Omega multimode microplate reader; substrates were added using the microplate reader injectors and data were normalized by total protein content. PPARγ transcriptional activity is expressed as the change in relative light units/µg of protein compared to luciferase alone.

### Statistical Analysis

Data are reported as mean values ± SD of *n* = 3 independent experiments per group, with 3 repeats per experiment. All results were statistically analyzed by one-way analysis of variance (ANOVA) for repeated measures. Differences between groups were assessed by Bonferroni’s multiple comparisons’ test. Values of *p* ≤ 0.05 were considered of statistical significance. Analytical procedures were performed using the scientific statistic software GraphPad Prism 8 (GraphPad Scientific, San Diego, CA, USA).

## Results

### AEA-Induced Neuroprotection Against Energy Deficiency and Excitotoxicity Relies on PPARγ and CB_1_ Receptors Activation

**Fig. 1:**
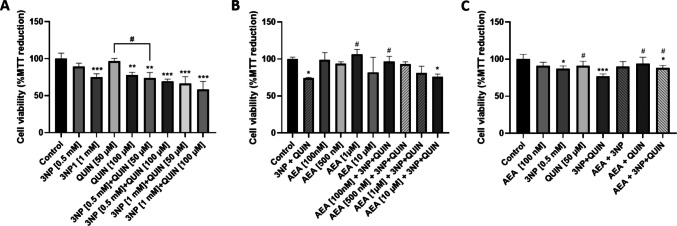
Characterization of the neurotoxic model on cell viability**.** (**A**) MTT viability assays in neurons treated with increased concentrations of 3NP and QUIN separately and in co-treatment for 24 h. (**B**) Increased concentrations of AEA administered for 6 h prior to treatment with 3NP + QUIN for an additional 24 h. (**C**) Six-hour pretreatment with AEA followed by 3NP or QUIN, separately. All data are presented as means ± SD of n = 3 experiments per group, with 4 repeats per experiment for the MTT assay, and 3 for the ATP assay. Statistical significance was calculated using a one-way ANOVA followed by Bonferroni’s multiple comparisons test. (*) Denotes differences *vs.* the control. (#) Denotes differences *vs.* 3NP + QUIN. * p = 0.0394, ** *p* = 0.0455, ** p = 0.0021 *** *p* < 0.0001, ^#^* p* = 0.0160, ^###^* p* < 0.0001

**Fig. 2:**
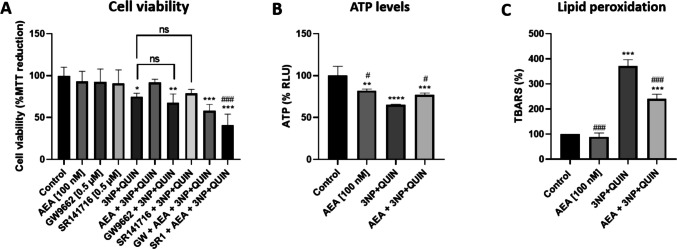
Effects of AEA on cell viability, ATP levels and lipid peroxidation in neurons exposed to 3NP + QUIN**.** (**A)** MTT viability assay in neurons treated with 100 nM AEA 3NP + QUIN and/or the PPARγ and CB_1_ receptor antagonists GW9662 and SR141716, respectively. (**B)** ATP levels assessment. (**C)** Lipid peroxidation assay. All data are presented as means ± SD of n = 3 experiments per group, with 4 repeats per experiment for the MTT assay, and 3 for the ATP assay. For the assessment of lipid peroxidation, a total of 3 repetitions were quantified in the same experiment. Statistical significance was calculated using a one-way ANOVA followed by Bonferroni’s multiple comparisons test. (*) Denotes differences *vs.* the control. (#) Denotes differences *vs.* 3NP + QUIN. *^,#^* p* = 0.0152, ** p = 0.0023, ***^,###^* p* ≤ 0.001, ***^,###^* p* ≤ 0.0001

To evaluate the toxicity of 3NP and QUIN alone and when added together, cortical primary neurons were treated with increased concentrations of each neurotoxin. After 0.5 mM 3NP + 50 μM QUIN co-treatment, viability was reduced to a greater extent than when administered separately, suggesting an additive effect on their toxicity (Fig. 1A). Neurons were pretreated with increased concentrations of AEA 6 h before injury, and concentration–response characterization showed that AEA 100 nM was the optimal concentration to evoke neuroprotective effects after 0.5 mM 3NP + 50 μM QUIN treatment for 24 h (Fig. 1B). When evaluating AEA protection against 3NP or QUIN toxicity separately, we observed that AEA prevented only 3NP damage. In turn, QUIN alone had no effect on reducing cell viability, while AEA prevented again the combined toxic effect of 3NP + QUIN (Fig. 1C). Both the PPARγ antagonist GW9662 and the CB_1_ receptor antagonist SR141716 reverted the effect of AEA in preventing the reduction of cell viability induced by 3NP + QUIN, suggesting that both nuclear and plasma membrane receptors contribute to the neuroprotective effects mediated by AEA (Fig. 2A). AEA alone had no effect on cell viability.

Co-treatment of neuronal cells with 3NP + QUIN also decreased total ATP levels by 40% compared to the control group, while AEA partially attenuated this effect, resulting in a significant recovery compared to the 3NP + QUIN treatment group (Fig. 2B). Notably, the treatment of cells with AEA alone decreased ATP levels by about 20% compared to the control group. Lipid peroxidation, assessed using the TBARS assay, showed that 3NP + QUIN treatment increased TBARS signal by 2.5-fold compared to the control group, and this effect was attenuated in the presence of AEA compared to the 3NP + QUIN group by 45% (Fig. 2C), thus confirming its antioxidant capacity [25].

### AEA Increases Mitochondrial Biogenesis and Mass

**Fig. 3:**
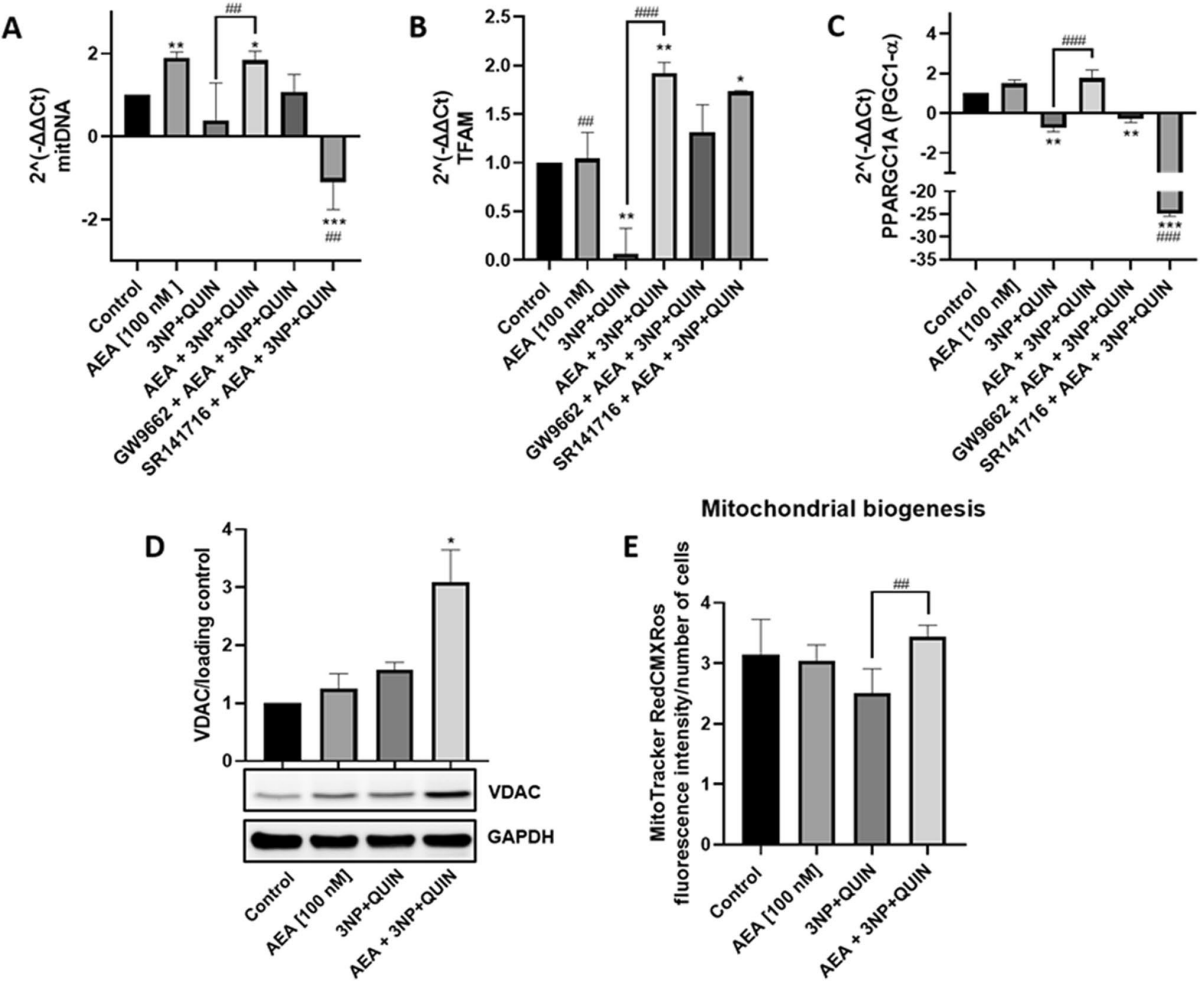
Effect of AEA on the expression of mitochondrial markers in neuronal cells exposed to 3NP + QUIN**.** (In **A-C)** The relative expression of mtDNA and genes TFAM and PPARGC1A (PGC1-α protein) was quantified by qPCR and normalized against actin as a housekeeping gene (2^(-ΔΔCt). (In **D**) Western blot assay for VDAC and densitometry. (In **E)** Quantification of fluorescence intensity of MitoTracker RedCMXRos. All data are present means ± SD of n = 3 experiments per group, with 2 repeats per experiment. Statistical significance was calculated by one-way ANOVA followed by Bonferroni’s multiple comparisons test. (*) Denotes differences *vs.* the control. (#) Denotes differences *vs.* 3NP + QUIN. * p = 0.0166, **^,##^* p* ≤ 0.0094, ***^.###^* p* ≤ 0.0001

Mitochondrial DNA copy number (mitDNAcn) was assessed by quantitative real-time PCR (qPCR) as an indicator of mitochondrial mass (Fig. 3A). While AEA alone increased mitDNAcn by almost two-fold, the 3NP + QUIN condition decreased it by about 50%. Neurons pretreated with AEA and subsequently exposed to 3NP + QUIN exhibited a significant increase in mitochondrial mass, approximately threefold higher than that observed in cells treated with 3NP + QUIN alone. Treatment with SR141716, but not with GW9662, prevented the AEA-induced increase in mitDNAcn by around 150%, suggesting the involvement of the CB_1_ receptor in the effect of AEA.

The relative expression of TFAM and PPARGC1A genes (the latter coding for PGC1-α) was quantified by qPCR (Figs. 3B and 3 C, respectively). While the combination of 3NP + QUIN inhibited the TFAM and PGC-1α expression by 90% and 60% compared to the control group, respectively (Fig. 3B-C), neurons pretreated with AEA increased the TFAM expression and prevented the decrease in PGC-1α expression. Both GW9662 and SR141716 attenuated the effect of AEA on TFAM expression in neurons exposed to 3NP + QUIN (Fig. 3B), suggesting that CB_1_ and PPARγ receptors are involved in the AEA-induced mitochondrial biogenesis pathway. GW9662 moderately inhibited the effect of AEA on PGC-1α expression in neurons treated with 3NP + QUIN, whereas SR141716 exerted a stronger inhibitory effect on AEA-induced responses, suggesting differential regulatory roles of PPARγ and CB_1_ receptors in this context (Fig. 3C).

The voltage-dependent anion channel (VDAC), a protein located in the outer mitochondrial membrane, is involved in the regulation of mitochondrial function and neuronal energy metabolism by regulating the exchange of ions and metabolites between mitochondria and cytosol. While treatment with 3NP + QUIN led to a moderate increase in VDAC expression (~ 30%), pretreatment with AEA resulted in a substantially greater upregulation, with VDAC levels nearly threefold higher (Figs. 3D). AEA alone did not modify the basal levels of VDAC expression. These results suggest that a compensatory upregulation of mitochondrial mass induced by toxin-induced mitochondria dysfunction is primed by the presence of AEA. Although this may indicate an increase in protein regulation, it could also be aimed at restoring mitochondrial function in response to underlying dysfunction [36].

To characterize in more detail the increase in mitochondrial mass, fluorescent detection was carried out using MitoTracker Red CMXRos [32]. While the 3NP + QUIN condition decreased Mitotracker signal compared to the control group (25%), neurons pretreated with AEA induced a recovery in the staining signal compared to the 3NP + QUIN group (Fig. 3E). Collectively, these findings suggest that AEA promotes mitochondrial biogenesis pathways to enhance mitochondrial mass, which may contribute to mitigating the damage induced by 3NP + QUIN.

### AEA Induces Regulation of Mitochondrial Morphology and Dynamics

**Fig. 4:**
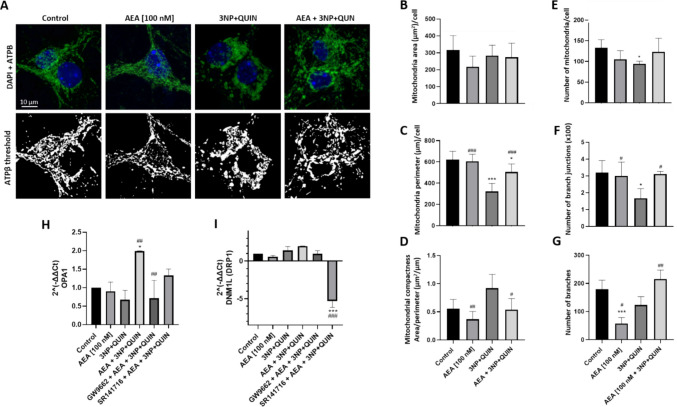
Effects of AEA on 3NP + QUIN-induced morphological changes in mitochondrial network in neuronal cells**.** Fluorescent labeling of the ATPβ protein was used to specifically visualize mitochondria, obtaining a microscopic image. (**A**) Immunofluorescence for ATPβ, and DAPI. 63X objective, 5X zoom. (**B-G**) Image processing and analysis for quantification of mitochondrial morphology parameters per neuron in fixed cells analyzed with the FIJI Mitochondria Analyzer plugin. (**B**) Mitochondrial area. (**C**) Mitochondrial perimeter. (**D**) Mitochondrial compactness. (**E**) Number of mitochondria, (**F**) Number of mitochondrial branches, (**G**) Number of mitochondrial branch junctions per cell. (**H-I)** The relative expression of OPA1 and DNM1L (DRP1 protein) was quantified by qPCR and normalized against actin as a housekeeping gen. All data are presented as means ± SD of n = 3 experiments per group. The fluorescence of 8 neurons per group was quantified. Statistical significance was calculated by one-way ANOVA followed by Bonferroni’s multiple comparisons test. (*) Denotes differences *vs.* the control. ** p = 0.0012. (#) Denotes differences from 3NP + QUIN. * p = 0.0165, ** p ≤ 0.0087, ^##^* p* = 0.0043, ***^,###^* p* ≤ 0.0001

ATPβ, also known as the beta subunit of ATP synthase, frequently used to study mitochondrial morphology due to its specific location within the inner mitochondrial membrane, was detected by immunofluorescence to assess mitochondrial morphology [37, 38]. Quantification of ATPβ fluorescence intensity revealed no significant changes in response to either AEA or 3NP + QUIN treatments alone when compared to the control group. In contrast, combined treatment with AEA and 3NP + QUIN resulted in a significant 75% increase in ATPβ levels (data not shown). Further morphological assessment of neurons comprised fluorescent staining and threshold of ATPβ (Figs. 4A).

Mitochondrial branching is important for the efficient distribution of proteins within the network, and a larger number of branches and junctions allows more efficient network connectivity. Although no changes were detected in mitochondrial area of neurons exposed to the different treatments (Fig. 3B), morphological analysis using the FIJI mitochondria analyzer plugin showed decreased mitochondrial perimeter by 50%, number of branches (70%) and branch junctions (50%), and an increase in mitochondrial compactness (50%) (Figs. 4C-4G) in neurons treated with 3NP + QUIN compared to the control group. In contrast, neurons pretreated with AEA and subsequently exposed to 3NP + QUIN exhibited a restoration across all assessed mitochondrial morphological parameters relative to the 3NP + QUIN group (Figs. 4B-4G). These results suggest that AEA induced network remodeling in the presence of toxic insults.

### AEA Increases the Expression of Genes Associated with Mitochondrial Dynamics

To determine the participation of AEA in the regulation of mitochondrial dynamics, the relative expression of OPA1 gene (which participates in mitochondrial fusion) and DNM1L gene (coding for DRP1, involved in mitochondrial fission) was quantified by qPCR and normalized against actin as a housekeeping gene using the 2^(-ΔΔCt) method (Figs. 4H, I, respectively). While the 3NP + QUIN group did not show significant changes in the expression of these genes compared to the control group (Figs. 4H, I), pretreatment with AEA in neurons exposed to 3NP + QUIN led to a significant, approximately twofold increase in OPA1 expression (Fig. 4H). Notably, while both GW9662 and SR141716 reversed the effects of AEA on OPA expression in neurons exposed to 3NP + QUIN (Fig. 4H), SR141716 disproportionately decreased DRP1 expression in AEA plus 3NP + QUIN treated cells (Fig. 4I), which in turn might suggest a more prominent role of CB_1_ than PPARγ. AEA alone did not affect these endpoints. These results suggest that AEA is involved in the regulation of mitochondrial dynamics at the fusion level, supporting an active role of DRP1 and OPA1 in several physiological processes [39–41].

### AEA Delays the Increase in Calcium Levels and Preserves Mitochondrial Membrane Potential

**Fig. 5:**
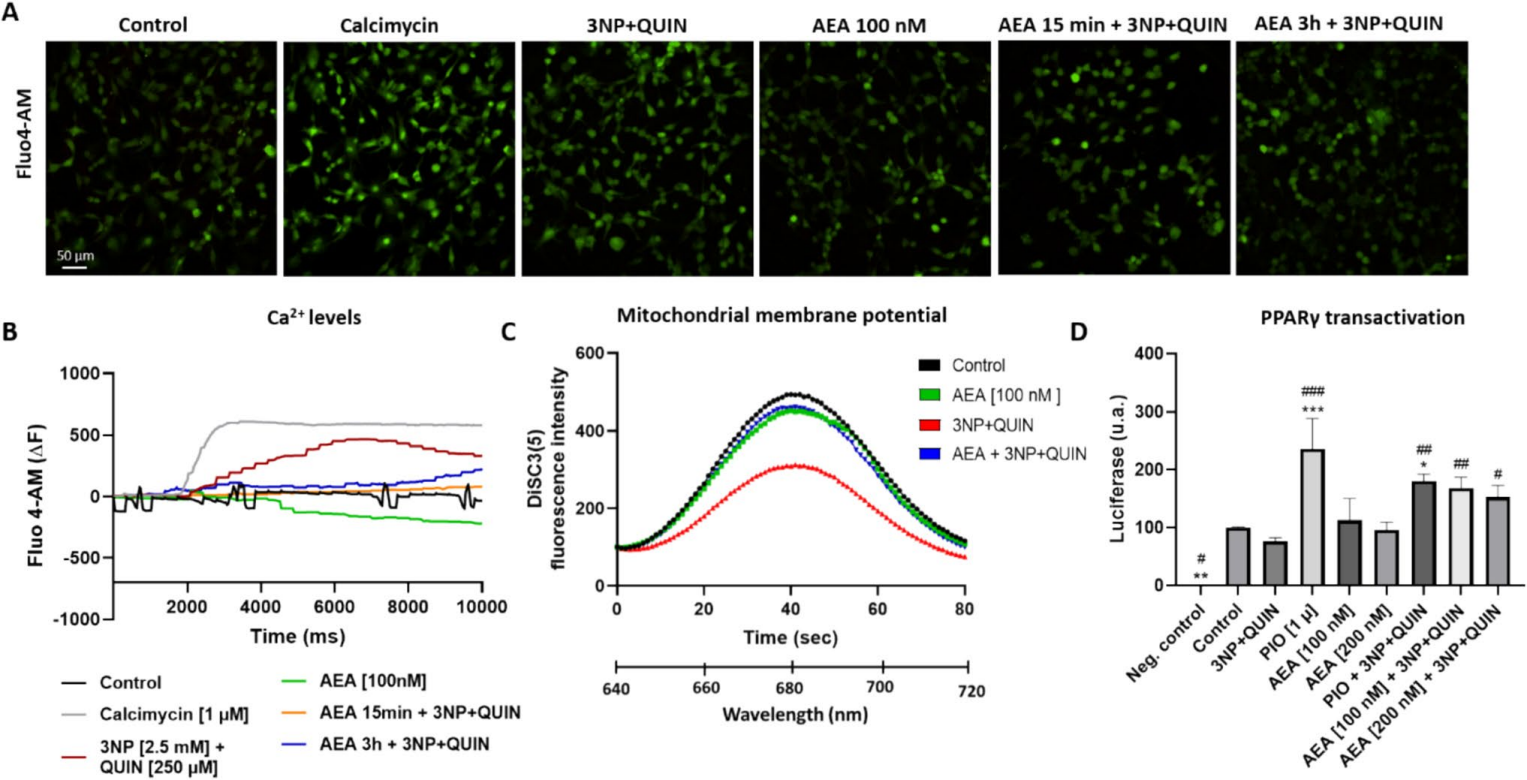
Effect of AEA on changes in calcium levels, mitochondrial membrane potential and PPAR γ transactivation in 3NP + QUIN exposed neuronal cells (**A**) Neurons exposed to different treatments: Control, Calcimycin (1 µM), 3NP (2.5 mM) + QUIN (250 µM), AEA (100 nM), AEA (100 nM) for 15 min + 3NP + QUIN, 100 nM AEA for 3 h + 3NP + QUIN; all in cells loaded with Fluo4-AM as a calcium marker. (**B**) Quantification of changes in calcium levels, detected by Fluo4-AM fluorescence, after the different treatments. (**C**) Fluorescence intensity of the DiSC3(5) probe after 6 h of treatment with AEA and an additional 24 h of treatment with 3NP + QUIN in digitonin-permeabilized cells; n = 3 experiments per group, with 1 measurement per experiment. (**D**) Luminescent luciferase signal as an indicator of PPAR***γ*** transcriptional activity in cortical neurons nucleofected with the Gal4-PPARγ-luciferase construct after 5 h of pretreatment with AEA followed by 1 h of exposure to toxins. All data are presented as means ± SD. Statistical significance was calculated by one-way ANOVA followed by Bonferroni’s multiple comparisons test. (*) Denotes differences *vs.* the control. (#) Denotes differences from 3NP + QUIN. * p = 0.0394, ** p = 0.0043, *** p < 0.0001, ^#^* p* = 0.0377, ^##^* p* = 0.0071, ^###^* p* < 0.0001

Cortical neurons were loaded with Fluo4-AM and treated with 3NP + QUIN and AEA to investigate the role of intracellular Ca^2+^ levels in neurotoxicity (Fig. 5A-5B). Cortical neurons responded immediately with increased intracellular calcium levels upon the addition of calcimycin [1 µM], a calcium ionophore used as a positive control. Treatment with 3NP + QUIN also increased intracellular calcium but in a slower manner. Pretreatment for 3 h with AEA in part decreased the toxin-induced calcium increase levels. Intracellular calcium level dynamics are shown as additional videos in Supplementary Fig. 1 (Figure S1).

Mitochondrial membrane potential (Δ*ΨM,* MMP) is a key indicator of proper mitochondrial function, and its alteration represents a key marker of impaired mitochondrial function [42, 43]. We used DiSC3(5) as a fluorescent cationic dye to measure Δ*ΨM* (Fig. 5C). AEA alone slightly decreased MMP. As expected, the 3NP + QUIN treatment led to a consistent reduction in mitochondrial membrane potential (ΔΨM) compared to the control group. In contrast, pretreatment of neurons with (AEA) prior to 3NP + QUIN exposure effectively restored ΔΨM to baseline levels.

### AEA induces PPARγ transactivation in degenerating neurons

To evaluate the effect of AEA on PPARγ transcriptional activity, GAL4 transactivation-based assays were used. Results showed an AEA-induced increase in PPARγ transactivation in neurons incubated under toxic conditions, with AEA at concentrations ranging 100 nM and 200 nM and in the presence of 3NP + QUIN, while AEA alone did not increase PPARγ transcriptional activity in control neurons (Fig. 5D). Treatment with 3NP + QUIN alone did not significantly change PPARγ transactivation. These results demonstrate that AEA has the capacity to activate PPARγ but only under degenerating conditions, when this activation is required.

### AEA Preserves Oxygen Consumption in Neurons Exposed to Toxic Insults

**Fig. 6:**
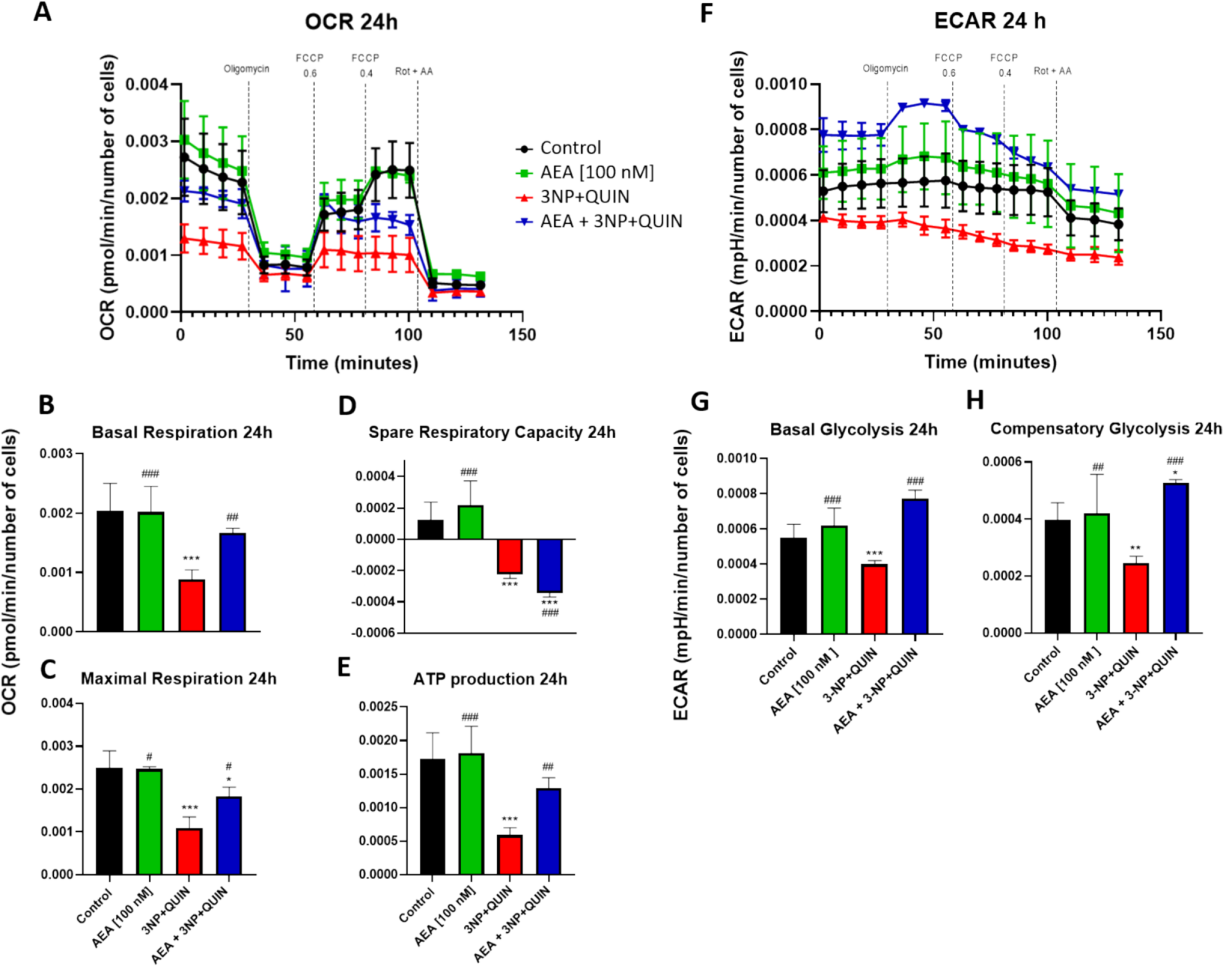
Effects of AEA (6 h of treatment) on markers of alterations in mitochondrial function (OCR and ECAR) in 3NP + QUIN (24 h) exposed neuronal cells**.** OCR (in **A**) and ECAR (in **F**) were measured using the Seahorse Bioscience Extracellular Flux Analyzer. Dashed lines indicate incubation of cells with the indicated compounds. Using the OCR and ECAR data, graphs of basal respiration (**B**), maximal respiration (**C**), spare respiratory capacity (**D**) and ATP production (**E**), as well as basal glycolysis (**G**) and compensatory glycolysis (**H**) were obtained. All data are presented as means ± SD. Statistical significance was calculated by one-way ANOVA followed by Bonferroni’s multiple comparisons test. (*) Denotes differences *vs.* the control. (#) Denotes differences from 3NP + QUIN. * p = 0.0231, ^#^* p* = 0.0112, **^,##^* p* = 0.0026, ***^,###^* p* ≤ 0.0001

To evaluate changes in cellular energy metabolism, oxygen consumption rate (OCR) was assessed as an indicator of mitochondrial respiration, and the extracellular acidification rate (ECAR) was assessed as an indicator of glycolysis (Fig. 6), after 30 min or 24 h of toxins exposure. Treatment with toxins for 30 min did not induce changes in either OCR or ECAR (data not shown). In turn, OCR analysis after 24 h of toxins exposure (Fig. 6A) revealed that 3NP + QUIN induced a decrease in basal respiration (Fig. 6B), maximal respiration (Fig. 6C), spare respiratory capacity (Fig. 6D), and ATP production (Fig. 6E) compared to the control group. In contrast, cells pretreated for six hours with AEA exhibited significant recovery in basal respiration, maximal respiratory capacity, and ATP production compared to neurons treated with 3NP + QUIN alone (Figs. 6B, 6 C and 6E). AEA alone did not affect the basal mitochondrial functions. These data suggest that AEA may boost neuronal oxidative metabolism in the presence of neurodegenerative insults.

The ECAR data at 24 h (Fig. 6F) showed that the insult induced by 3NP + QUIN decreased basal glycolysis (Fig. 6G) and compensatory glycolysis (Fig. 6H) compared to the control group. Pretreatment of neuronal cells exposed to 3NP + QUIN with anandamide (AEA) fully restored these two endpoints relative to the group receiving the neurodegenerative insult alone (Figs. 6F, 6G, and 6H). Treatment with AEA alone did not significantly alter these endpoints compared to the control group.

## Discussion

ECB signaling exerts neuroprotective functions via multiple receptor pathways, highlighting its multitarget potential and unveiling complex mechanisms involved in pathological neuronal signaling. Cell viability assays, quantification of MTT reduction, and ATP levels showed that the 3NP + QUIN insult induces neuronal damage, interfering with mitochondrial metabolic activity. Results derived from the lipid peroxidation assay further implicate oxidative stress as a contributing factor of neurodegeneration, and that AEA attenuates this toxicity mechanism. QUIN induced neurodegeneration is mediated by the overactivation of NMDA receptors, resulting in enhanced calcium levels, energy deficit, oxidative stress and cell death [44]. In turn, the main toxic mechanism evoked by 3NP involves its intracellular incorporation into mitochondria and the inhibition of energy production, which can lead to ATP depletion while enhancing excitotoxicity [45, 46]. In addition, 3NP has been reported to increase intracellular calcium levels through NMDA receptor activation, thus contributing to cell damage [47]. Thus, the combined neurodegeneration induced by 3NP and QUIN co-treatment integrates mitochondrial dysfunction with excitotoxicity, generating a model of neurodegeneration characterized by significant decrease of cell viability that surpasses the effects of each toxin alone. Here, we demonstrate that AEA delayed the toxin-mediated intracellular Ca^2+^ levels during the early phase of exposure, thus preserving cellular homeostasis.

Treatment with AEA prevented both oxidative damage and the decreased viability in neurons at a concentration of 100 nM, in line with previous reports showing that AEA, at nanomolar concentrations, prevents neurodegeneration and hypoxic injury in a CB_1_ receptor dependent manner [48, 49]. Alternatively, high concentrations of AEA can be deleterious to neuronal survival in agreement with the classical biphasic actions of cannabinoids.

We observed that the antagonists GW9662 and SR141716, inhibited AEA neuroprotection, indicating that both PPARγ and CB_1_ receptors mediate the protective effects of this EC. Due to its lipophilic nature, AEA can readily diffuse across cell membrane to reach PPARγ receptors, and mitochondrial CB_1_ receptors (mitCB_1_). Notably, PPARγ receptor activity can result from direct interaction with cannabinoids, indirectly driven from membrane CB_1_ receptor signaling or from PPARγ interaction with AEA-derived metabolites [50]. Further supporting these concepts, our study provides evidence that AEA induces PPARγ transactivation under neurodegenerative conditions.

The decrease in mitDNAcn and mitotracker fluorescence in neurons exposed to the toxins, and their prevention by AEA, suggest that the neuroprotective effect of AEA is associated with increased mitochondrial mass. VDAC regulates mitochondrial function and neuronal energy metabolism [36]. Also, AEA increased VDAC expression in neurons treated with 3NP + QUIN, but not under control conditions, suggesting that AEA promotes the compensatory upregulation of mitochondrial biogenesis and function in response to mitochondrial dysfunction.

PGC1-α, the coactivator of the PPARγ receptor, is key for the induction of mitochondrial biogenesis by promoting the transcription of genes such as TFAM. Assessment by qPCR indicated that AEA increased the expression of genes encoding PGC1-α and TFAM proteins, and this effect was prevented by GW9662, thus linking the increase in mitochondrial mass to a PPARγ-dependent mitochondrial biogenesis mechanism. CB1 antagonism promoted a greater downregulation of mitDNA and the PPARGC1A gene that encodes the protein PGC1-α, than PPARγ antagonism, suggesting a prominent role of the CB1 receptor in AEA-induced mitochondrial biogenesis. This is consistent with studies showing the ability of CB1 to promote mitDNA and PGC-1α expression via CB1 [19], whereas Δ9-THCA, a double CB1 and PPARγ agonist efficiently upregulates PGC-1α expression [51]. Furthermore, corroborating earlier findings showing that CB1 signaling induces PPARγ transactivation [52, 53], our results also confirmed that the regulation of PPARγ by CB1 activity was associated with regulation of mitochondrial biogenesis induced by AEA.

Balanced mitochondrial fission and fusion dynamics are critical for maintaining mitochondrial bioenergetics and synaptic integrity in neurons. Inhibition of Drp1, a key mediator of mitochondrial fission, confers neuroprotective effects in both in vitro and in vivo models. An imbalance favoring excessive fission is frequently associated with neuronal degeneration observed in neurodegenerative diseases [54]. In contrast, OPA1 is an essential GTPase responsible for mitochondrial inner membrane fusion. Adenoviral mediated expression of OPA1 has been shown to restore in vitro mitochondrial function [55], while its deficiency hastens age-related deficits in learning and memory [56]. Mitochondrial fusion plays a protective role against neuronal stress by enabling the redistribution of mitochondrial contents and facilitating the exchange of mitochondrial components. This process supports cellular homeostasis, preserves mitochondrial function, and contributes to the delay of apoptotic signaling [57, 58]. Here we observed an increase in OPA1 gene expression in AEA-treated neurons exposed to 3NP + QUIN, and no significant change in DRP1 gene expression in neurons under the same conditions, suggesting that the neuroprotective effect of AEA is more dependent on mitochondrial fusion. The increase in OPA1 expression induced by AEA was inhibited by the PPARγ antagonist, but not by the CB1 antagonist, indicating the participation of PPARγ in the regulation of AEA-induced OPA1 transcription in a CB1-independent manner.

Although mitochondrial morphology in neurons exhibits substantial variability across subcellular compartments, namely the soma, dendrites, and axons [59], morphological alterations typically result from changes in fission, fusion rates and mitochondrial membrane potential. AEA pretreatment decreased mitochondrial compactness, while increasing the number of branches and branch junctions. The increase in branches after AEA pretreatment is consistent with increased fusion and the ability of mitochondria to generate elongated branched network. We hypothesize that the lack of significant changes in mitochondrial number may be due to increased fusion. Mitochondrial compaction in the soma also decreased with AEA pretreatment, suggesting decreased death signaling [60].

Mitochondrial membrane potential (ΔΨm) is a key indicator of respiratory chain deficits, proton leak due to membrane damage, or enhanced activation of the mitochondrial permeability transition pore (mPTP) [61]. 3NP + QUIN treatment led to decreased ΔΨm, OCR and ECAR. Pretreatment with AEA partially attenuated the decline in ΔΨm and OCR, while significantly enhancing ECAR compared to the control group, 24 h after injury. These findings align with previous reports demonstrating that antioxidant compounds can mitigate mitochondrial damage induced by 3NP and QUIN. Such protective effects are thought to be mediated through the activation of endogenous antioxidant pathways, particularly the Nrf2/ARE signaling axis, in addition to their direct capacity to neutralize reactive oxygen species as free radical scavengers [62, 63], and these mechanisms were also inherent to AEA treatment [25].

Neurons have limited metabolic flexibility, relying primarily on oxidative phosphorylation (OXPHO) for ATP production and utilizing lactate supplied by astrocytes as an additional energy source [64]. Nonetheless, neurons can positively regulate glycolysis even when experiencing mitochondrial respiration stress [65]. ECAR is the value of the extracellular lactic acid production rate, which correlates with glycolysis. AEA significantly increased the neuronal ECAR rate following 24 h of exposure to neurodegenerative stimuli. Furthermore, a marked peak in ECAR was detected upon administration of oligomycin, an inhibitor of mitochondrial ATP synthase, indicating a shift toward glycolytic compensation. These findings suggest that AEA promotes an adaptive metabolic response in neurons, potentially enhancing cellular resilience under conditions of mitochondrial dysfunction. Notably, neurons treated with toxins without AEA did not show an increase in ECAR. Thus, the observed effects support a role for AEA as a modulator of the metabolic shift under pathological conditions. This ability to influence cellular energy reprogramming positions AEA as a promising candidate for further investigation in the context of neurodegenerative disease and mitochondrial dysfunction.

We have also observed that AEA alone, in healthy neurons, produced metabolic and mitochondrial alterations, such as decreased ATP, decreased ΔΨm and oxygen consumption. This observation suggests that the neuroprotective effects of AEA may be conditional, manifesting primarily under stress- or damage-induced states, while eliciting distinct responses in neurons under basal conditions. Such context-dependent effects are particularly relevant given the differential expression patterns of cannabinoid receptors observed in pathological versus physiological states. The reduction of mitochondrial activity, membrane potential and ATP production may be explained by direct binding of AEA to F_o_F_1_ ATP synthase inhibiting this activity but not the hydrolase activity [66]. It has also been reported that AEA decreases the mitochondrial membrane potential while increasing the fluidity of the outer mitochondrial membrane at micromolar concentrations when directly incorporates to membrane [67]. Furthermore, mtCB_1_ activation has been shown to decrease complex I activity, oxygen consumption, and ATP levels; however, these effects of AEA on mitochondria do not promote neuronal death mechanisms [68–70], thus suggesting that they remain at a regulatory level, not at a toxic level. In this context, investigating the role of mitochondrial CB_1_ receptors (mtCB_1_) in mediating the AEA-induced effects reported here warrants particular attention.

## Conclusion

Collectively, our novel findings demonstrate that anandamide AEA confers neuroprotection via activation of CB_1_ receptors and the induction of PPARγ-mediated transcriptional pathways. These effects were associated with increased mitochondrial biogenesis and enhanced mitochondrial function, thereby supporting neuronal metabolic activity and viability. The combined neurodegenerative insult of 3NP and QUIN employed in this model significantly reduced cell viability and elevated oxidative stress markers, effectively recapitulating key metabolic, mitochondrial, and morphological disturbances characteristic of neurodegenerative diseases. These findings contribute to elucidating the diversity of neuroprotective mechanisms induced by the ECS and hence of relevance for neurodegenerative diseases.

## Supplementary Information

Below is the link to the electronic supplementary material.

Supplementary file1 (PDF 170 KB)

Supplementary file2 (PPTX 30.8 MB)

Supplementary file3 (PDF 347 KB)

## Data Availability

The data that support the findings of this review are available from the corresponding author upon reasonable request.
