# Supplementary material for: Anandamide-Induced Neuroprotection of Cortical Neurons Relies on Metabolic/Redox Regulation and Mitochondrial Dynamics
Source: Mol Neurobiol. 2025 Nov 24;63(1):153. doi: 10.1007/s12035-025-05514-z (PMC12641045; doi:10.1007/s12035-025-05514-z)
Supplement: Supplementary file 1 — Supplementary file1 (PDF 170 KB) [file 12035_2025_5514_MOESM1_ESM.pdf]

Supplementary Material

Table 1. Forward and reverse sequences of the primers used for the detection of the genes DNM1L (DRP1), OPA1, TFAM, PPARGC1A (PGC1-α), ACTB (β-Actin) and mtDNA used in the qPCR assays.

Figure S1. **15-second videos showing changes in Ca^{2+} currents induced by different treatments in real time with the calcium label Fluo4-AM.** Neurons were exposed to different treatments: Control, Calcimycin (1 μM), 3NP (2.5 mM)+QUIN (250 μM), AEA (100 nM), AEA (100 nM) for 15 min + 3NP+QUIN, 100 nM AEA for 3 hours + 3NP+QUIN; all in cells loaded with Fluo4-AM as a calcium marker. Quantification of changes in calcium currents was carried out after the different treatments.

Supplementary Material

Table. S.1.

Genes	Forward primer	Reverse primer
**mtDNA**	TCGCCTACTCCTCAGTTAGC	TCCGTTCGTAGTTGGAGTTTG
**DNM1L (DRP1)**	GGGCACTTAAATTGGGCTCC	TGTATTCTGTTGGCGTGGAAC
**OPA1**	TCACCTCTGCGTTTATTTGAAGA	GGGTAGAACGGGAGGAAAGG
**TFAM**	TCCCCTCGTCTATCAGTCTTGT	CCACAGGGCTGCAATTTTCC
**PPARGC1A (PGC1-α)**	TGAAAGGGCCAAACAGAGAGA	TAAATCACACGGCGCTCTTCA
**ACTB (β-Actin)**	CATTGCTGACAGGATGCAGAAGG	TGCTGGAAGGTGGACAGTGAGG

Figure S.1.

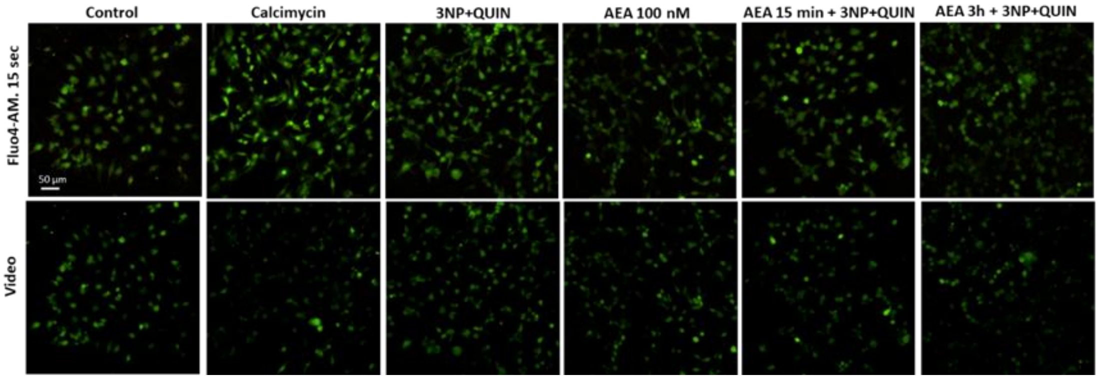
Control
Calcimycin
3NP+QUIN
AEA 100 nM
AEA 15 min + 3NP+QUIN
AEA 3h + 3NP+QUIN
Fluo4-AM. 15 sec
Video
50 µm
