# Supplementary material for: Anandamide-Induced Neuroprotection of Cortical Neurons Relies on Metabolic/Redox Regulation and Mitochondrial Dynamics
Source: Mol Neurobiol. 2025 Nov 24;63(1):153. doi: 10.1007/s12035-025-05514-z (PMC12641045; doi:10.1007/s12035-025-05514-z)
Supplement: Supplementary file 2 — Supplementary file2 (PPTX 30.8 MB) [file 12035_2025_5514_MOESM2_ESM.pptx]

## Slide 1
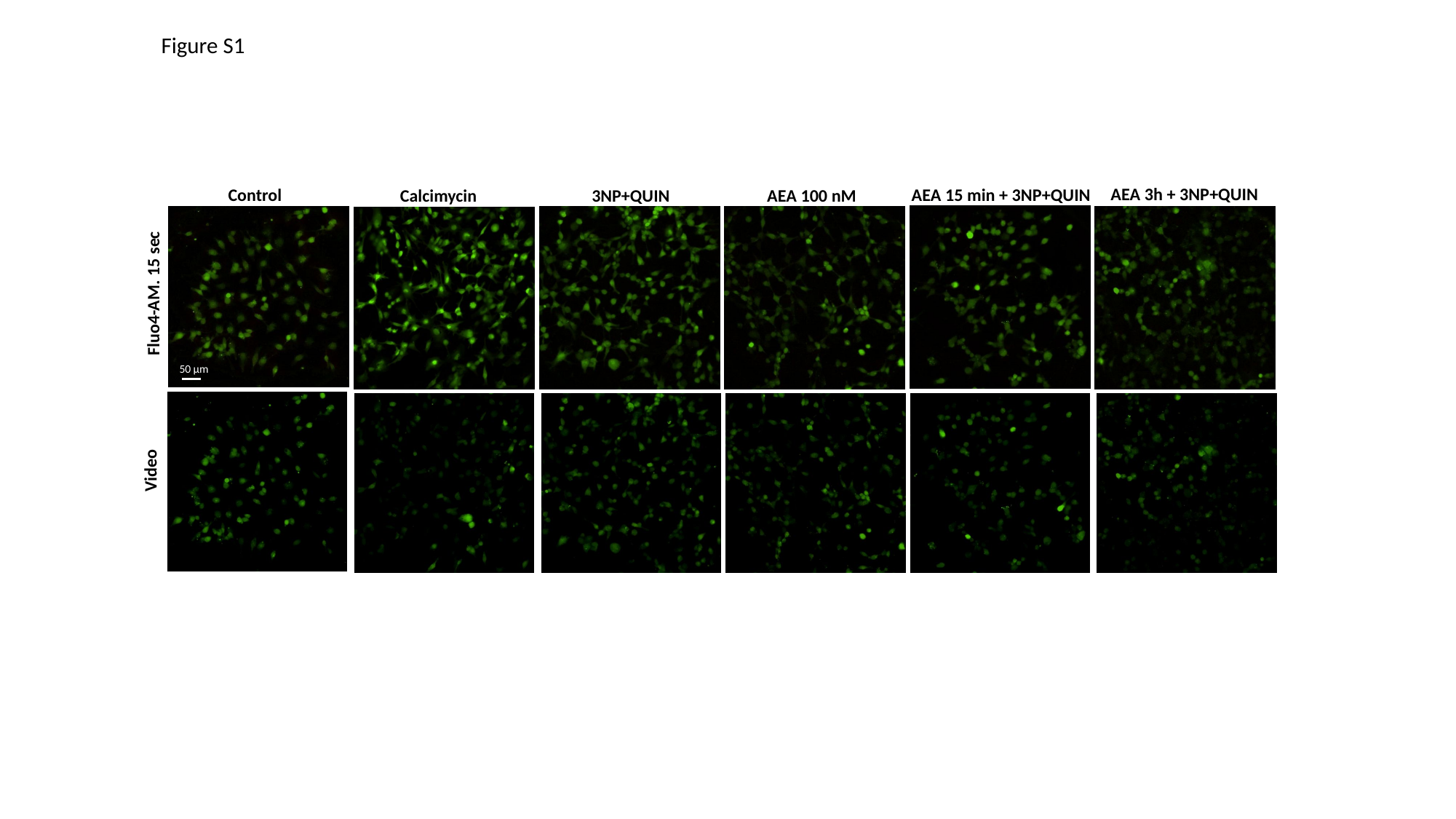

Figure S1
Calcimycin
AEA 3h + 3NP+QUIN
Control
AEA 15 min + 3NP+QUIN
3NP+QUIN
AEA 100 nM
Fluo4-AM. 15 sec
50 μm
50 μm
Video
