# Supplementary figures and images for: Anandamide-Induced Neuroprotection of Cortical Neurons Relies on Metabolic/Redox Regulation and Mitochondrial Dynamics
Source: Mol Neurobiol. 2025 Nov 24;63(1):153. doi: 10.1007/s12035-025-05514-z (PMC12641045; doi:10.1007/s12035-025-05514-z)

Complete gels of Figure 3D

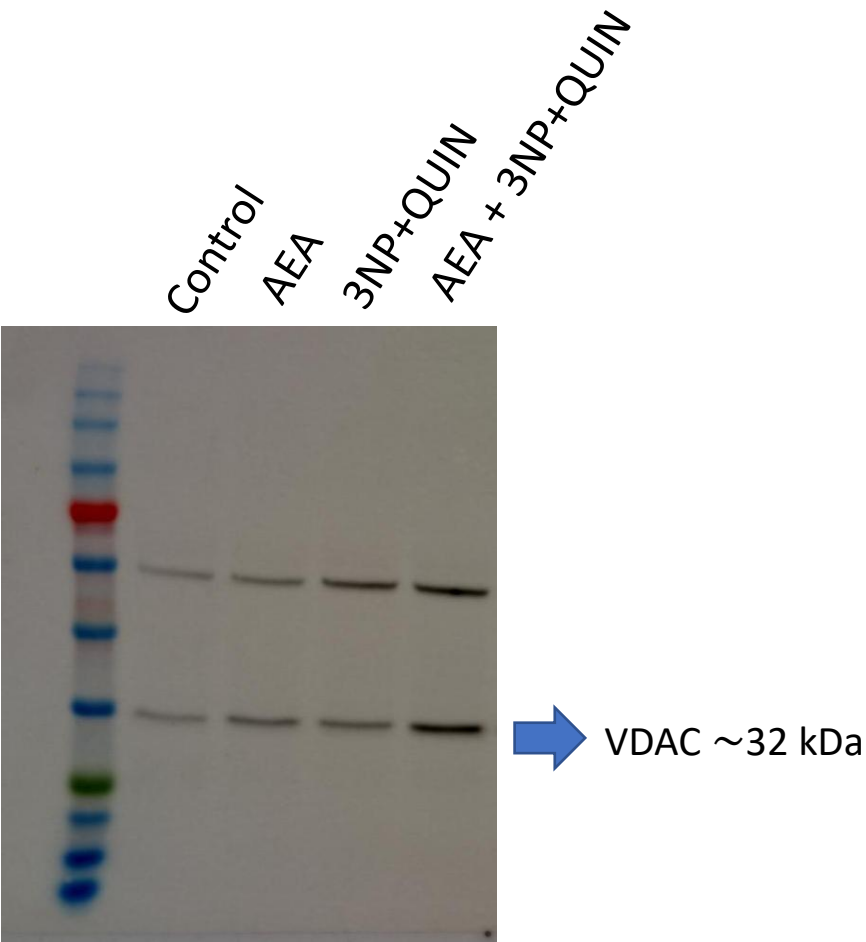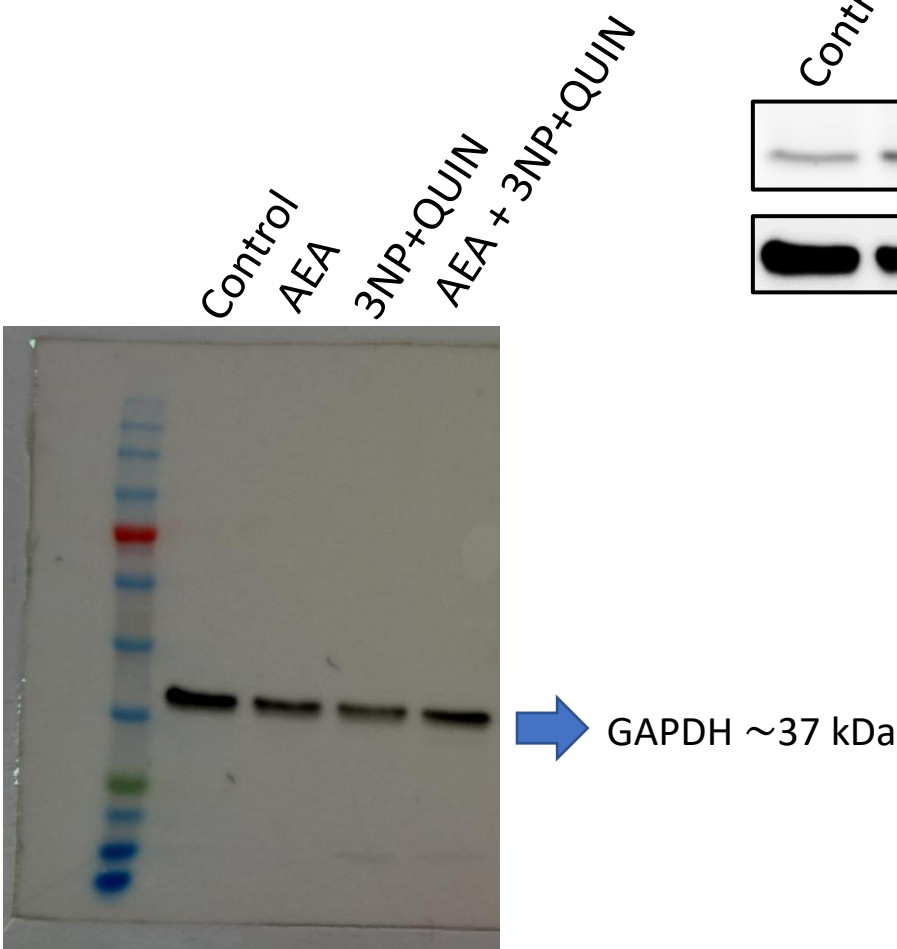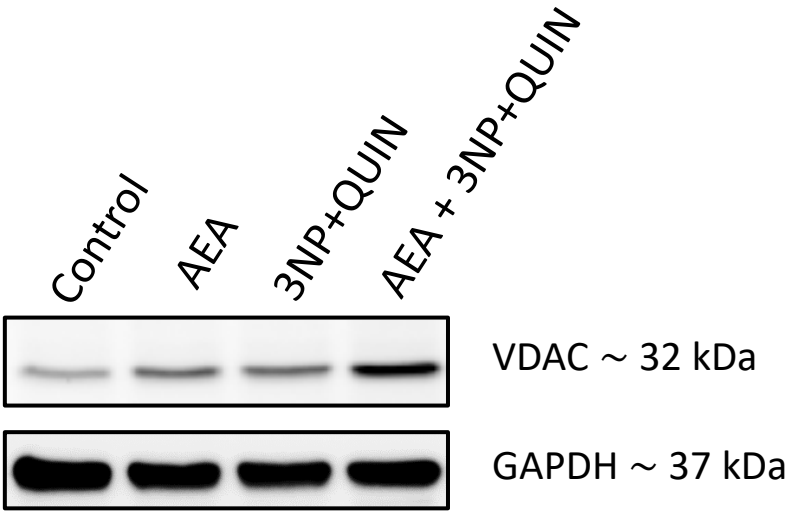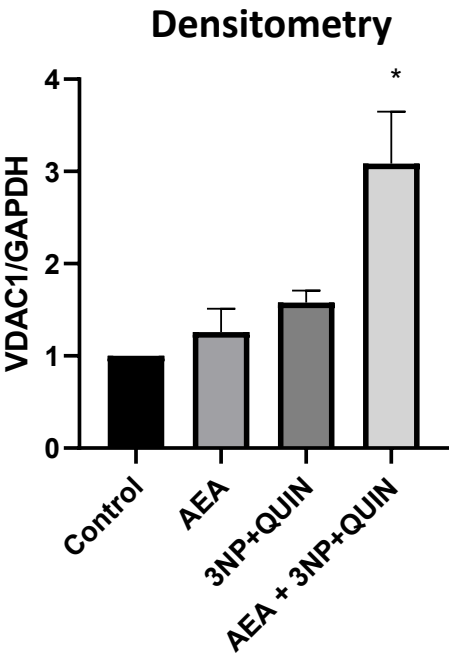

## Second repetition

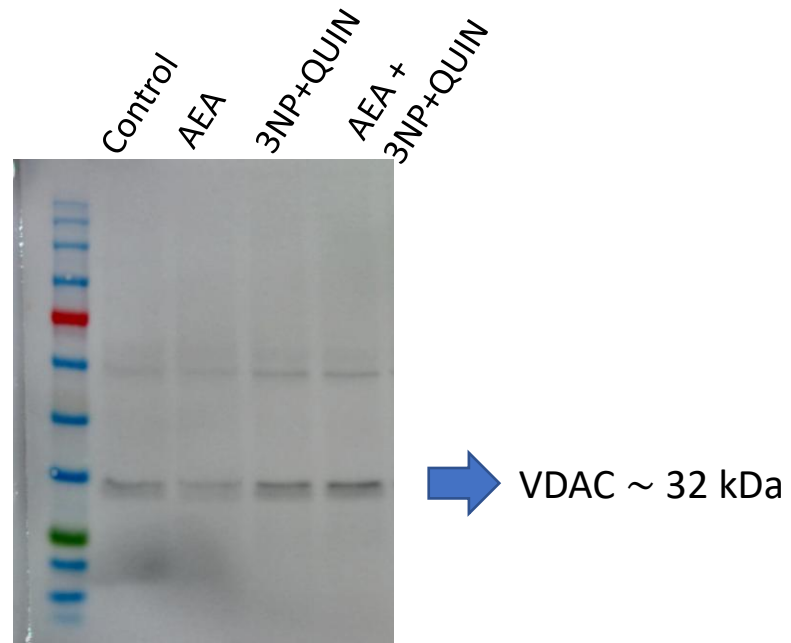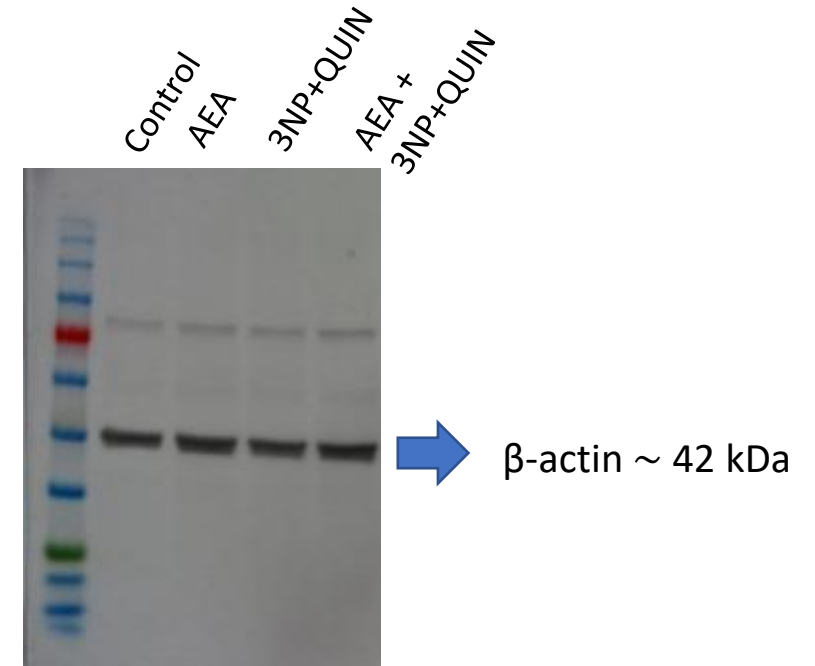

## Third repetition

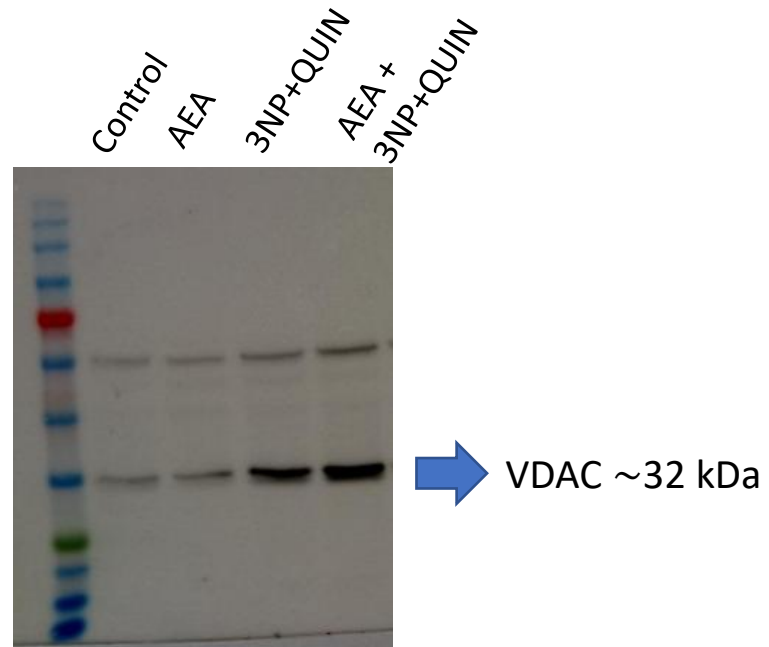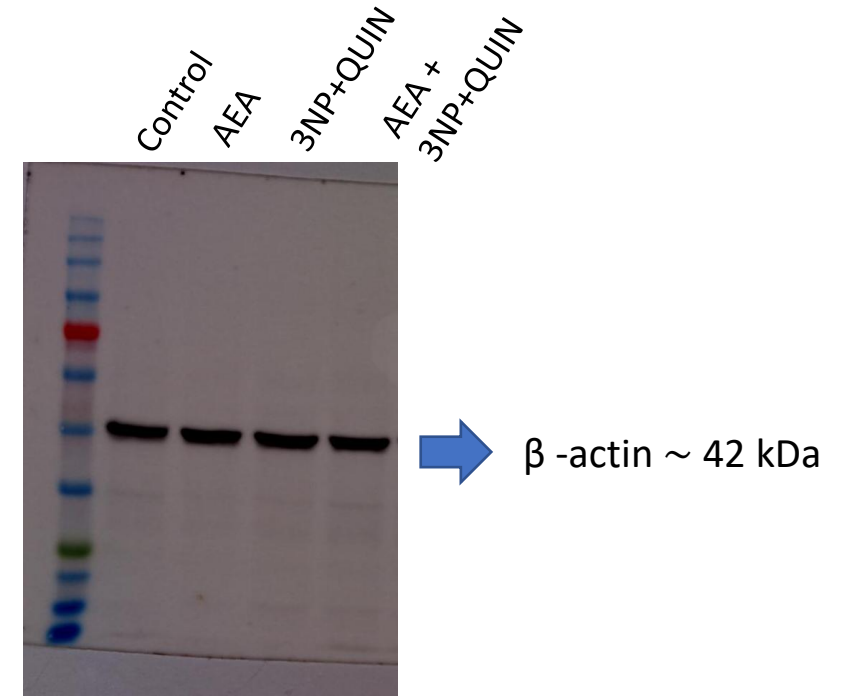

Supplement: Supplementary file 3 — Supplementary file3 (PDF 347 KB) [file 12035_2025_5514_MOESM3_ESM.pdf]
